# Phosphorylated Nitrones—Synthesis and Applications

**DOI:** 10.3390/molecules30061333

**Published:** 2025-03-16

**Authors:** Iwona Rozpara, José Marco-Contelles, Dorota G. Piotrowska, Iwona E. Głowacka

**Affiliations:** 1 Bioorganic Chemistry Laboratory, Faculty of Pharmacy, Medical University of Lodz, Muszynskiego 1, 90-151 Lodz, Poland; iwona.rozpara@umed.lodz.pl; 2 Laboratory of Medicinal Chemistry, Institute of General Organic Chemistry (CSIC), Juan de la Cierva 3, 28006 Madrid, Spain; jlmarco@iqog.csic.es

**Keywords:** nitrones, phosphonates, synthesis, spin-trapping agents, biological activity

## Abstract

Phosphorylated nitrones belong to an important class of compounds with several applications, such as their therapeutic potency to reduce oxidative stress or as spin-trapping agents. This review covers available synthetic methods for the preparation of both non-cyclic and cyclic phosphorylated nitrones, including the possibilities of the modification of structures with selected functional groups, as well as examples of their application. As reported, the incorporation of diethoxyphosphoryl function into the structure of PBN and DMPO resulted in obtaining their phosphorylated analogs, i.e., *N*-benzylidene-1-diethoxyphosphoryl-1-methylethylamine *N*-oxide (PPN) and 5-(diethoxyphosphoryl)-5-methyl-1-pyrroline-*N*-oxide (DEPMPO), respectively, both forming spin adducts of improved stability in comparison to the reference non-phosphorus nitrones. Moreover, antioxidant and neuroprotective activity observed in the group of phosphorylated nitrones makes them promising candidates for therapeutics.

## 1. Introduction

**Figure 1:**
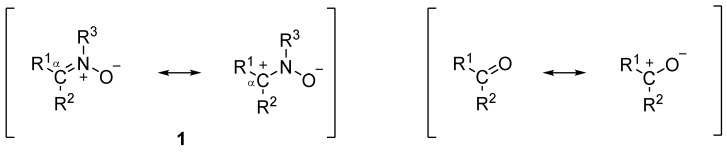
Mesomeric effect of nitrones and ketones.

Nitrones **1** (Figure 1) [1] represent a class of compounds with a variety of applications, including their reactivity as substrates in organic chemistry [1,2,3] and their therapeutic usefulness in the treatment of oxidative stress [4,5,6,7,8] or electron spin traps [9,10]. The term “nitrone” appeared in 1916 for the first time. This shortcut was created by combining the words nitrogen and ketone [11], due to the structural analogy, i.e., mesomeric effect in both nitrones and ketones (Figure 1) [1]. Moreover, reactions with nucleophiles occur via attacking the C(sp^2^) atom of nitrone function analogously to the reactivity of the carbonyl group [12].

**Figure 2:**
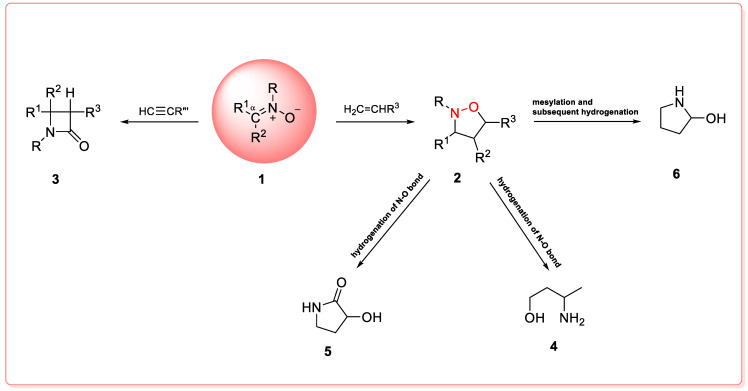
Examples of the application of nitrones in organic synthesis.

1,3-Dipolar cycloaddition (1,3-DC) is an important transformation in organic chemistry leading to five-membered heterocycles [1,2,3]. When nitrone is used as a dipole in cycloaddition with alkenes, isoxazolidines **2** [1,2,3] are formed, while the Cu-catalyzed Kinugasa reaction of nitrone with alkynes produces β-lactams **3** [13,14] (Figure 2), and up to even two (for β-lactams) or three stereogenic centers (for isoxazolidines) can be generated. The stereoselectivity of the 1,3-DC reaction is dependent on the chirality of nitrones and dipolarophiles, as well as the catalyst used in the respective reactions [15]. Furthermore, isoxazolidine derivatives **2** are useful intermediates in organic chemistry and could be easily transformed into 1,3-aminoalcohols **4** [16], α-hydroxy-γ-lactams **5** [17], and hydroxylated pyrrolidine **6** [18] (Figure 2). In addition to the chemical applications of isoxazolidines in various chemical transformations, several examples of biologically active agents have been reported in this class of compounds, including isoxazolidine derivatives exhibiting antibacterial [19,20], antiviral [21,22], and anticancer activities [23,24], among others. The above-mentioned strategies depicted in Figure 2 have also been applied to the preparation of phosphorylated compounds such as phosphorylated isoxazolidines [21,22,25,26,27,28,29,30,31,32,33,34,35] and β-lactams [36,37,38], phosphoproline [18,39] and pyrrolidinone analogs [39], and phosphonate analogs of homoserine [40] and 4-hydroxyglutamic acid [41], as well as other α-amino-β-hydroxyphosphonates [42].

**Figure 3:**
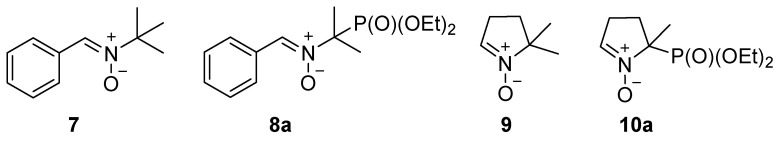
Structures of nitrones **7**, **8a**, **9**, and **10a**.

Nitrones have found applications not only in organic chemistry. They are able to react and stabilize with free radicals, and for this reason, they have found applications as agents for the detection of highly unstable free radicals. Free radicals are chemical species that contain unpaired electrons [43,44]. They can be divided into reactive oxygen species (ROS) containing an oxygen atom and reactive nitrogen species (RNS) containing a nitrogen atom [45]. They are identified as products of cellular metabolism and play a crucial role in the living system [46]. For example, free radicals are signaling molecules [43,47] and Ca^2+^ channel stimulators [48,49] and are involved in immunological defense [50]. Unfortunately, when an overproduction of ROS and RNS occurs and the antioxidant system is impaired, oxidative and nitrosative stress appear [51]. Oxidative and nitrosative stress have been associated with cardiovascular diseases [52,53] (such as atherosclerosis [54], ischemia/reperfusion injury [55], and hypertension [56]), neurodegenerative diseases [57], such as Parkinson’s disease [58] and Alzheimer’s diseases [59], and cancer [46,51]. Free radicals are very reactive [43,44], with their detection being difficult. To detect ROS and RNS in the biological system, EPR spin trapping was used. This method is based on the reaction of a “spin trap” with free radicals and the analysis of the resulting more stable product, which is called a “spin adduct” [9,10]. Due to their high persistence and specificity towards free radicals, nitrones are among the most useful “spin traps” and can be classified as non-cyclic nitrones, such as PBN (**7**) (Figure 3) [10] and its analog PPN (**8a**) (Figure 3) [60], or cyclic nitrones, such as DMPO (**9**) (Figure 3) [10] and its analog DEPMPO (**10a**) (Figure 3) [10,61,62].

**Figure 4:**
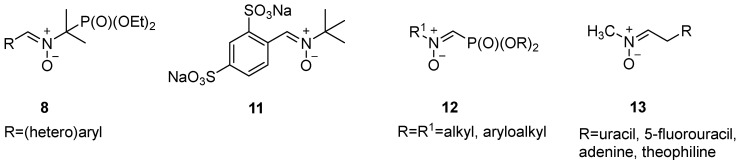
Structures of compounds **8** and **11–13**.

Free radical scavengers, due to their ability to react with free radicals, can be used as potential therapeutics for many diseases to which nitrosative and oxidative stress may contribute. In 1985 and 1986, the first experiments on the pharmacological properties of PBN (**7**) (Figure 3) were carried out in animal models. PBN (**7**) has been found to protect against death after endotoxin shock, as well as shock trauma [4,5]. In addition, PBN (**7**) (Figure 3) demonstrated neuroprotective activity in stroke [63,64]. These studies initiated intensive work on the pharmacological properties of other nitrones. One of the most promising compounds was 2,4-disulfophenyl-*N*-*tert*-butylnitrone (**11**, NXY-059, currently known as OKN-007) (Figure 4), which exhibited a neuroprotective effect in experimental ischemia studies [6,7]. Unfortunately, nitrone **11** (Figure 4) appeared ineffective as a stroke neuroprotectant in a large clinical trial [65,66]. On the other hand, nitrone **11** (Figure 4) has been demonstrated to have an impact on reducing the growth of glioblastoma [67,68,69,70,71,72]. Recently, the antioxidant and neuroprotective properties of phosphorylated PPN-type nitrones **8** (Figure 4) [73] and *N*-substituted *C*-(dialkoxyphosphoryl)nitrones **12** (Figure 4) have been demonstrated [74]. The antioxidant and neuroprotective activity of nitrones **13** (Figure 4) has also been shown to be higher than that of PBN (**7**) (Figure 3) [8].

Numerous reviews on nitrones have been published over the years [1,75,76], however there was no comprehensive report on phosphorylated nitrones. This review covers literature reports on the synthesis of phosphorylated nitrones and their applications from the early 1990s to the present. Phosphorylated nitrones are useful synthons in organic chemistry since they react with alkenes to form functionalized isoxazolidines [77,78] as well as with alkynes to give β-lactams [36,37,38]. Among the various isoxazolidine cycloadducts, nucleos(t)ide analogs with an isoxazolidine moiety that mimics a natural sugar are of particular interest due to their observed antiviral and anticancer properties [21,22,30,31,32,33,34]. Phosphorylated nitrones are also excellent spin traps for detecting free radicals using EPR spin trapping [60,61,62]. Moreover, due to the capacity of nitrones to react with free radicals, they may be useful as potential agents in the treatment of diseases to which oxidative and nitrosative stress may contribute [73,74].

## 2. Synthesis of Nitrones

Phosphorylated nitrones comprise three groups of compounds: *N*-substituted *C*-phosphorylated nitrones, “non-cyclic” phosphorylated nitrones, and cyclic phosphorylated nitrones.

### 2.1. N-Substituted C-Phosphorylated Nitrones

**Figure 5:**
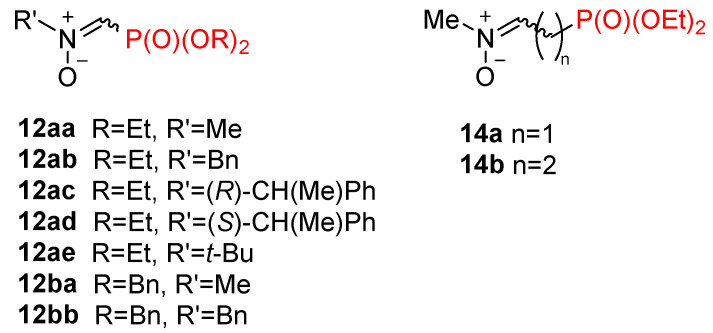
Structures of compounds of **12aa-ae**, **12ba**,**bb**, and **14a**,**b**.

*N*-substituted *C*-phosphorylated nitrones are a class of compounds having the phosphoryl group located directly at the *C* atom of nitrone function [30,35,74,77], i.e., *N*-substituted *C*-(dialkoxyphosphoryl)nitrones **12aa-ae** and **12ba-bb** (Figure 5) [74,77], or via an additional alkyl linker, i.e., (diethoxyphosphorylalkyl)nitrones **14a-b** (Figure 5) [30,35].

#### 2.1.1. N-Substituted C-(Dialkoxyphosphoryl)Nitrones 12

**Scheme 1:**
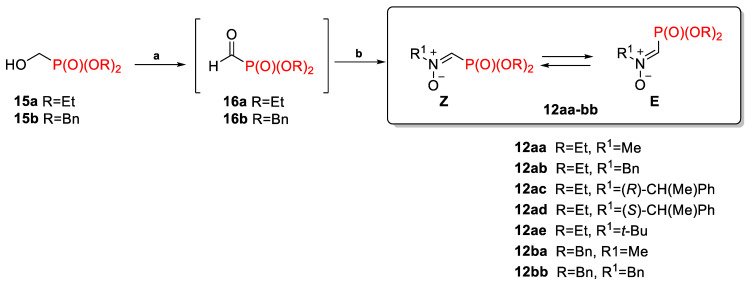
Synthesis of nitrones **12aa-bb**. Reagents and conditions: (a) (COCl)_2_, DMSO, −78 °C; (b) R’NHOH, r.t., 20–87%.

The Swern oxidation of diethyl (hydroxymethyl)phosphonate **15a** or dibenzyl (hydroxymethyl)phosphonate **15b** led to the in situ formation of the respective formylphosphonates **16a-b**, which were subsequently reacted with corresponding hydroxylamines to give nitrones **12aa-bb** (Scheme 1) [18,74,77]. Most of the nitrones **12** exist as an equilibrium mixture of *E*/*Z* isomers in CDCl_3_, and only **12ad** existed as a *Z* isomer; however, for all of them, the *Z* isomer is preferred in DMSO-d_6_ [18,74,77].

**Scheme 2:**
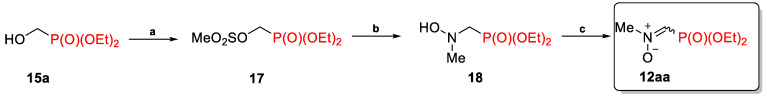
Synthesis of nitrone **12aa**. Reagents and conditions: (a) MeSO_2_Cl, CH_2_Cl_2_, Et_3_N, −10 °C, then r.t., 2 h, 90%; (b) MeNHOH, Et_3_N, reflux, 5 h, 50%; (c) MnO_2_, CH_2_Cl_2_, r.t., 18 h, 60%.

Romeo and co-workers reported an alternative methodology for the preparation of nitrone **12aa** based on the conversion of the (hydroxymethyl)phosphonate **15a** into mesylate **17** and its subsequent reaction with the *N*-methylhydroxylamine. The oxidation of derivative **18** with MnO_2_ resulted in the formation of nitrone **12aa** (Scheme 2). In this strategy, the formation of unstable formylphosphonate is avoided; however, the total yield of the transformations is noticeably lower (27%) compared to the original strategy via the Swern oxidation of **15** [34].

#### 2.1.2. N-Substituted C-(Diethoxyphosphorylalkyl)Nitrones 14

**Scheme 3:**
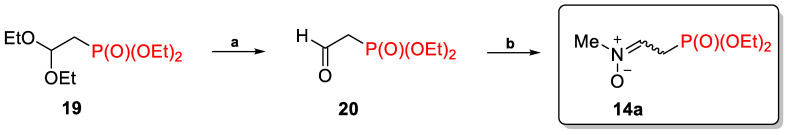
Synthesis of nitrone **14a**. Reagents and conditions: (a) Amberlist-15, acetone, r.t., 18 h, 95%; (b) MeNHOH×HCl, Et_3_N, toluene, r.t., 3 h, 95%.

For the synthesis of *N*-methyl *C*-(diethoxyphosphorylmethyl)nitrone **14a**, diethylphosphonoacetaldehyde diethyl acetal **19** was hydrolyzed to diethyl (2-oxoethyl)phosphonate **20** [30,79] and then reacted with *N*-methylhydroxylamine hydrochloride. After purification by column chromatography, nitrone **14a** was isolated as a 15:85 mixture of *E*/*Z* isomers and in a high total yield (75%) (Scheme 3) [30].

**Scheme 4:**
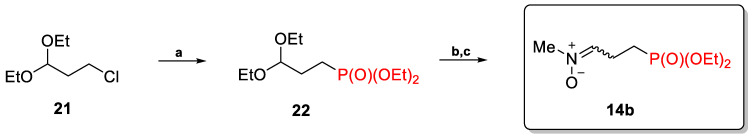
Synthesis of nitrone **14b**. Reagents and conditions: (a) (EtO)_3_P, reflux, 12 h; (b) 10% HCl, 90 °C, 2 h; (c) MeNHOH×HCl, Et_3_N, CH_2_Cl_2_, r.t., 5 h, 85%.

An analogous strategy was applied to the synthesis of nitrone **14b** (Scheme 4). Diethyl (3-oxopropyl)phosphonate diethyl acetal (**22**) obtained from 3-chloropropionaldehyde diethyl acetal (**21**) via the Arbuzov reaction with triethyl phosphite was hydrolyzed to diethyl 2-formylethylphosphonate which was then reacted with *N*-methylhydroxylamine hydrochloride to form *N*-methyl *C*-(diethoxyphosphorylethyl)nitrone (**14b**) as a 1:6 mixture of *E*/*Z* isomers [35].

### 2.2. “Non-Cyclic” Phosphorylated Nitrones

**Figure 6:**
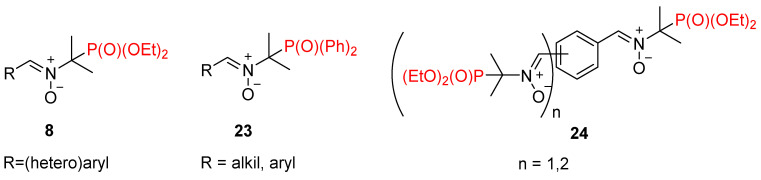
Structures of nitrones **8**, **23**, and **24**.

“Non-cyclic” phosphorylated nitrones were designed as the analogs of PBN (**7**) (Figure 3). In contrast to *N*-substituted *C*-phosphorylated nitrones **12** and **14** (Figure 5), they contain phosphoryl groups located in the substituent at the nitrogen atom of the nitrone function. These types of nitrones include PPN-type nitrones **8** [60,73,80,81,82,83], nitrones **23** [84] having a diphenylphosphinyl group, and poly(phosphorylated)nitrone **24** (Figure 6) [85].

#### 2.2.1. PPN-Type Nitrones 8

**Scheme 5:**
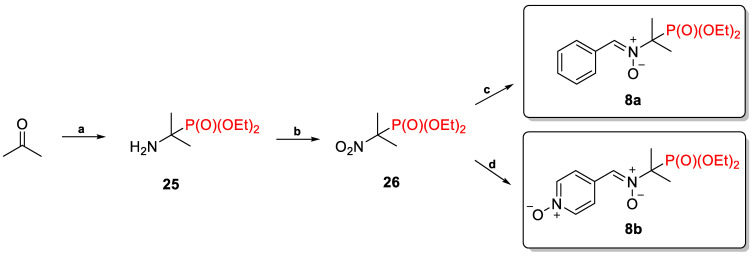
Synthesis of nitrones **8a** and **8b**. Reagents and conditions: (a) NH_3_, HP(O)(OEt)_2_; (b) KMnO_4_; (c) AcOH, EtOH, Zn, benzaldehyde, 30%; (d) Zn, NH_4_Cl, EtOH, 4-formylpyridine *N*-oxide, 27%.

Nitrone PPN **8a** and its analog **8b** were synthesized from acetone, which was treated with diethyl phosphite under a flow of ammonia to afford aminophosphonate **25**. Then, the oxidation of **25** with KMnO_4_ gave nitrophosphonate **26**. The reduction of **26** with zinc followed by a reaction with benzaldehyde or 4-formylpyridine *N*-oxide resulted in nitrone PPN **8a** or 4-PyOPN **8b** but with a low yield (not exceeding 30%) (Scheme 5) [60].

**Scheme 6:**
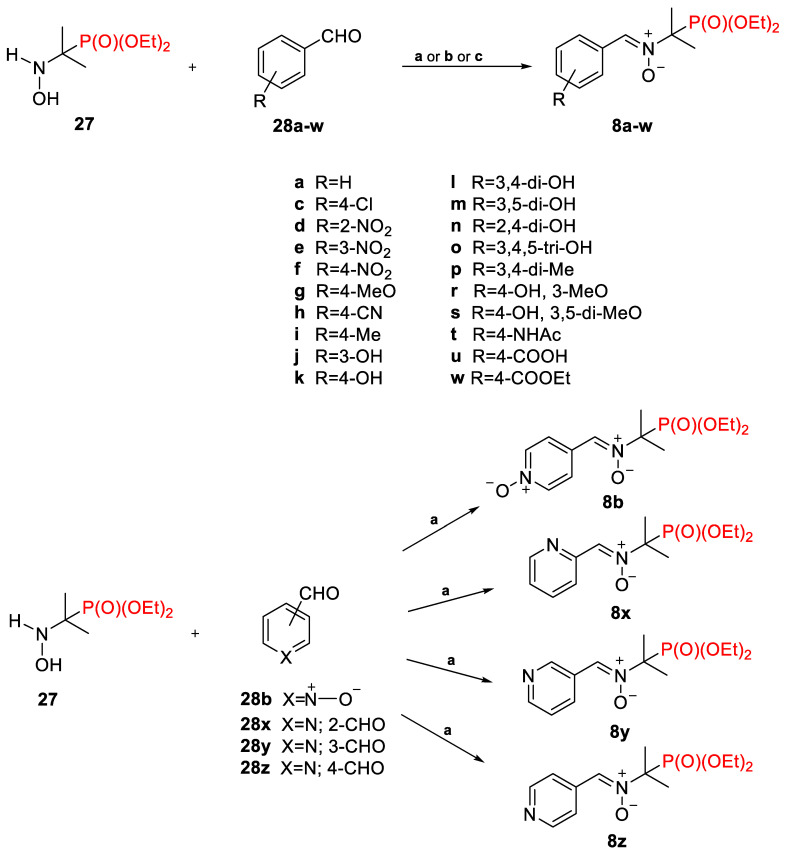
Synthesis nitrones **8a-z** from diethyl [1-(hydroxyamino)-1-methylethyl]phosphonate (**27**). Reagents and conditions: (a) EtOH, 55 °C, 3 h, ~90%; (b) EtOH, 60 °C, 22 h, 93%; (c) THF or DCE, MgSO_4_, 100 °C, 4 h, 35–70%.

Later on, the procedure for the preparation of **8a** and **8b,** as well as several PPN analogs **8c-z,** was improved by isolating the hydroxyloaminophosphonate **27**, formed as a product of the nitrophosphonate **26** reduction, and its recrystallization from pentane [73,80,81,82,86,87]. Then, the condensation of diethyl [1-(hydroxyamino)-1-methylethyl]phosphonate (**27**) with corresponding aldehydes **28a-z** gave nitrones **8a-z** (Scheme 6) [73,80,81,82,86,87,88].

**Scheme 7:**
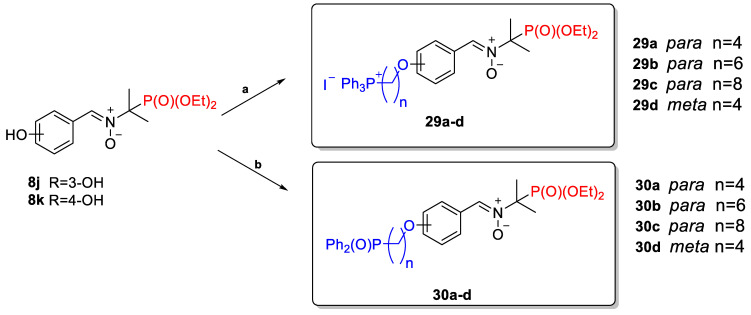
Synthesis of nitrones **29a-d** and **30a-d**. Reagents and conditions: (a) K_2_CO_3_, MeCN, rt, 30 min, then (iodoalkyl)triphenylphosphonium iodide **31a-c**, MeCN, 60 °C, 12 h, 25–40%; (b) NaH, MeCN, rt, 30 min, then (iodoalkyl)triphenylphosphonium iodide **31a-c**, 40 °C, 12 h, 32–64%.

The group of PPN-type nitrones was extended to include mito-PPNs **29a-d** functionalized as triphenylphosphonium salts (mitochondria-targeted “non-cyclic” phosphorylated nitrones) and nitrones having a diphenylphosphinyl group **30a-d** in the aromatic ring (non-mitochondria-targeted nitrones). Williamson’s reaction of respective phenol derivatives **8j** or **8k** with (iodoalkyl)triphenylphosphonium iodide **31a-c** in the presence of a respective base was applied to synthesize nitrones **29a-d** and **30a-d**. K_2_CO_3_ was used as a base for the synthesis of mito-PPNs **29a-d**, while the preparation of **30a-d** via Williamson’s reaction required a stronger base, such as sodium hydride (Scheme 7) [83]. As observed, the yield of the target nitrones **29a-d** and **30a-d** depends on the methylene chain length. Thus, the overall yields of the obtained nitrones decrease with chain length (**29a** vs. **29b** and **29c** as well as **30a** vs. **30b** and **30c**). Moreover, the mutual arrangement of respective groups in the aromatic ring present in the structures of the nitrone also affects the yield. Isomeric *para*-substituted derivatives **29a** and **30a** were obtained in slightly higher yields compared to the analogous *meta*-derivatives, **29d** and **30d**, respectively.

**Scheme 8:**
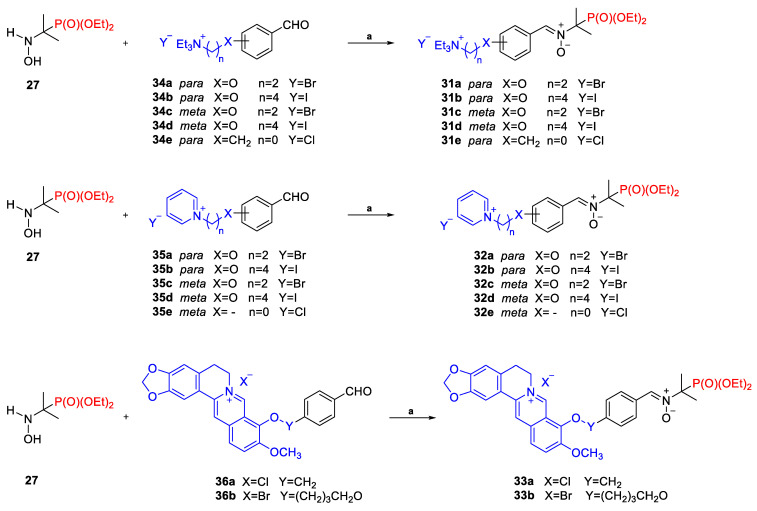
Synthesis of nitrones **31a-e**, **32a-e,** and **33a,b**. Reagents and conditions: (a) EtOH, 100 °C, 1h, microwave, 49–78% for **31a-e**, 53–93% for **32a-e**, 76% for **33a** and 73% for **33b**.

In continuation of the above-mentioned studies on nitrones **29** and **30**, a new class of mitochondrial-targeted nitrones **31**, **32,** and **33** (mito-PPNs) was designed (Scheme 8), obtained, and tested for their potential applications [89]. In these newly synthesized compounds, triethylammonium (**31a-e**) or pyridinium (**32a-e**) cations were installed instead of the triphenylphosphonium cation present in respective series **29**. Furthermore, in order to achieve expanded structural diversity, two additional mito-PPN nitrones (**33a**,**b**) containing a berberinium cation were obtained. While the synthetic methodology for the preparation of the previously obtained nitrones **29** and **30** was based on the respective functionalization of phenol-derived nitrones **8j** or **8k** (Scheme 7), newly designed compounds **31–33** were synthesized via a standard approach including the condensation of diethyl [1-(hydroxyamino)-1-methylethyl]phosphonate (**27**) with corresponding aldehydes **34**, **35**, or **36** as a key step (Scheme 8). The analogous synthetic strategy has already been successfully applied for the preparation of previously described nitrones **8a-z** as well (Scheme 6).

**Scheme 9:**
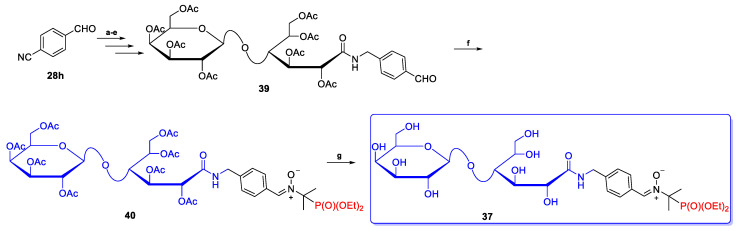
Synthesis nitrone **37**. Reagents and conditions: (a) *p*-TsOH, toluene, ethylene glycol, reflux, 9 h, then cooled at 20 °C, sodium bicarbonate, 90%; (b) lactobiono-1,5-lactone, LiAlH_4_, Et_2_O, 0 °C, 4 h, then r.t., 12 h, then EtOH and then a mixture of ethanol/water (1:1), 62%; (c) MeOH, TEA, reflux, 3 h, 85%; (d) acetic anhydride in pyridine (1:1), 0 °C, then r.t., 18 h, 53%; (e) *p*-TsOH, CH_3_CHO, 15 °C, 7 h, 86%; (f) hydroxylamine phosphonate **27**, THF, argon atmosphere, 40 °C, 76 h, 60%; (g) EtONa, EtOH, argon atmosphere, 20 °C, 20 min, 98%.

**Scheme 10:**
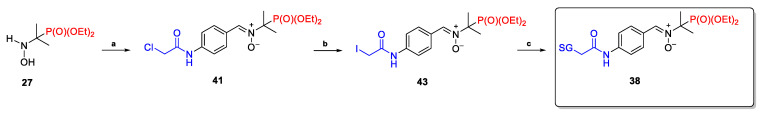
Synthesis nitrone **38**. Reagents and conditions: (a) 2-Chloro-*N*-(4-formylphenyl)acetamide, CHCl_3_, nitrogen atmosphere, reflux, 4 h, 91%; (b) NaI, EtOH, darkness, reflux, 80 h, 61%; (c) GSH, EtOH, K-phosphate buffer (0.1 M, pH 7.4), 25 °C, overnight, 100%.

The condensation of diethyl [1-(hydroxyamino)-1-methylethyl]phosphonate (**27**) with the respective aldehydes was a key step in the synthesis of nitrones **37** and **38**, derivatives of PPN **8a** functionalized with carbohydrate and glutathione, respectively (Scheme 9 and Scheme 10) [90,91]. The synthesis of glycosylated nitrone **37** was performed from *N*-(4-formylbenzyl)octa-*O*-acetyllactobionamide **39** prepared from 4-cyanobenzaldehyde (**28h**) in a multi-step reaction sequence (Scheme 9). The condensation of peracetylated aldehyde **39** with hydroxylamine phosphonate **27** was followed by hydrolysis of all acetyl groups in **40** to give the final nitrone **37** (Scheme 9) [91].

Glutathione-derived nitrone **38** was easily prepared in three steps from hydroxylamine phosphonate **27** which was condensed with 2-chloro-*N*-(4-formylphenyl)acetamide to give nitrone **41**. Nucleophilic substitution with an iodide anion led to the formation of derivative **42**, which was subsequently subjected to coupling with GSH to produce the designed nitrone **38** (Scheme 10) [90].

#### 2.2.2. Nitrones with Diphenylphosphinyl Group 23

**Scheme 11:**
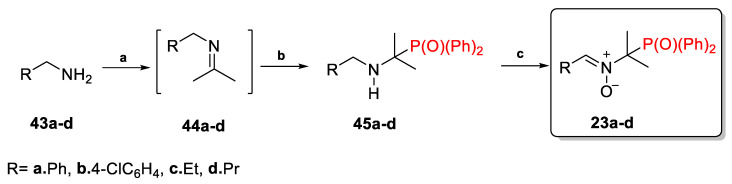
Synthesis nitrones **23a-d**. Reagents and conditions: (a) acetone, silica gel, m.w., 1 min; (b) diphenylphosphine oxide, silica gel, microwave irradiation, 1 min, 63–69%; (c) Oxone, acetone/NaHCO_3_, 0 °C, then r.t., 1 h, 34–47%.

Nitrones **23** with a diphenylphosphinyl group located at the nitrone atom of the nitrone function were efficiently synthesized by functionalization of the corresponding primary amines **43a-d** (Scheme 11). The reactions of amines **43a-d** with acetone led to the formation of the respective imines **44a-d**, which were then immediately reacted with diphenylphosphine oxide under microwave irradiation and in the presence of silica gel to produce secondary amines **45a-d**. Oxidation of the amines **45a-d** with Oxone was a key step to produce the nitrones **23a-d** (Scheme 11) [84].

#### 2.2.3. Poly(Phosphoryl)Nitrones 24

**Scheme 12:**
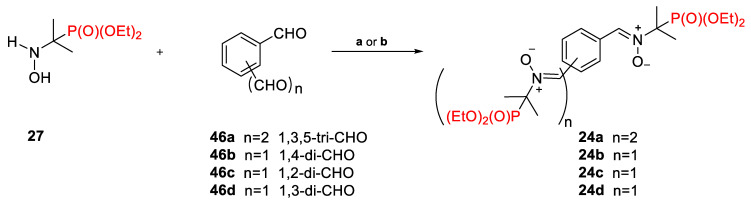
Synthesis nitrones **24a-d**. Reagents and conditions: (a) EtOH, 55 °C, 3 h, 64–89%; (b) benzene, 55 °C, 3 h, 65%.

A synthetic strategy based on the condensation of hydroxylamine **27** with corresponding aromatic polyaldehydes **46a-d** was applied for the preparation of compounds **24a-d** functionalized with two and three nitrone groups (Scheme 12), which was analagous to the methodology previously applied to obtain PPN-type nitrones **8** (Scheme 6) [85].

### 2.3. Cyclic Phosphorylated Nitrones

Cyclic nitrones with pyrrolidine units are of particular interest as spin traps for biological applications. For example, DMPO (**9**) was recognized to form characteristic adducts with a superoxide radical anion (O^•–2^) and a hydroxyl radical (^•^OH); however, its application as a probe for the generation of an oxyradical in biological systems is limited due to the suspected toxicity of the nitrone to biological tissue [92].

Several functionalized nitrones based on a pyrrolidine skeleton, including various phosphorus-containing analogs of DMPO (**9**) (Figure 3), were synthesized with the intention to study their properties and to understand the stability of the spin traps and the spin adducts in vivo. It was proven that DEPMPO (**10a**) (Figure 3), with the diethoxyphosphoryl function at C5, can trap and form a stable adduct for both ^•^OH and O^•–2^, giving the EPR spectra characteristics of each [61]. Later on, the structure of nitrone **10a** was further modified by introducing various alkyl/aryl groups in the phosphoryl function [61,93,94,95,96,97,98,99]. Moreover, the methyl group at C5 in the pyrrolidine ring in **10a** was replaced with other substituents [100,101,102]. Furthermore, additional substituents at C3 [103,104] and C4 [103,105,106,107] were also incorporated. Due to the large number of compounds, examples of nitrones are presented in this report according to the modification made to the structure of the model compound, namely DEPMPO (**10a**).

#### 2.3.1. Depmpo and Its Alkoxy-, Alkyl-, and Aryl-Phosphorylated Derivatives

**Scheme 13:**
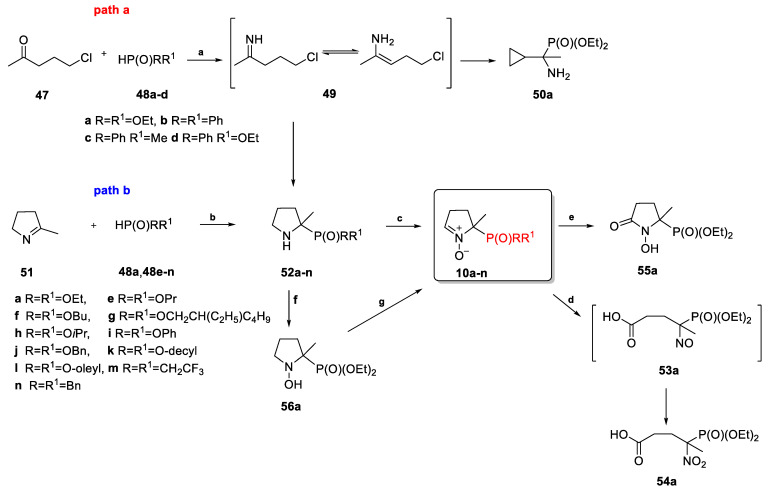
Synthesis of nitrones **10a-n**. Reagents and conditions: (a) NH_3_, EtOH, 50–60 °C, 3h; (b) r.t., 7–12 days (4 h for **49m**) 84–98%; (c) 2.2 eq. H_2_O_2_/Na_2_WO_4_, 0 °C, 48 h or *m*-CPBA, CHCl_3_, −10 °C, 1 h or Oxone, NaHCO_3_, acetone, r.t., 1 h or 2 eq. PSPO, CHCl_3_, 0 °C or 2 equiv DMD, acetone, 0 °C, 20 min; (d) m-CPBA, CHCl_3_, **53**: 14%, **54**: 8%, or Oxone, acetone, **53**: 22%, **54**: 9%; (e) 2 equiv DMD, acetone, 0 °C, 20 min, 5%; (f) 1 eq. DMD, acetone, 0 °C, 20 min, 75%; (g) Cu(OAc)_2_, MeCN, 30 min, 90%.

Several synthetic strategies for the preparation of DEPMPO (**10a**) and its analogs **10b-n** rely on the application of respective pyrrolidine derivatives **52a-n** in the key oxidation step (Scheme 13) [61,93,94,95,96,98,99,108].

Pyrrolidines **52**, required as key compounds in the above-mentioned strategy, were prepared using two different synthetic routes. The Kabachnik–Fields reaction of 5-chloropentan-2-one (**47**) and corresponding diethyl phosphite **48a** or phosphine oxides **48b-d** was applied for the preparation of **52a-d** (Scheme 13, path a) [61,94,95,96,97,107], while the reaction of 2-methyl-1-pyrroline (**51**) and corresponding phosphite derivative **48a** and **48e-n** was used for the preparation of **52a** and **52e-n** (Scheme 13, path b) [93,94,99,108].

Although 5-chloropentan-2-one (**47**) is widely used as a starting material in the synthesis of phosphorylated nitrone **10a**, the formation of diethyl (1-amino-1-cyclopropylethyl)phosphonate (**50a**) as a by-product was noted and explained as resulting from the imine–enamine tautomerization of intermediate **49** (Scheme 13, path a) [108].

Various oxidizing agents were used to oxidize compounds **52** to **10**, including *m*-CPBA [61,94,96,108], Na_2_WO_4_/H_2_O_2_ [93,94,108], Oxone [94,95,97,108], Davi’s reagents (PSPO), and dimethyldioxirane (DMD) [94,108]. Unfortunately, the formation of the over-oxidation product was observed when stronger oxidants (Oxone, *m*-CPBA, and excess of DMD) were used, namely **53**, **54**, and **55** (Scheme 13) [94,108]. However, when a stoichiometric amount of DMD was employed, hydroxylamine **56** was produced, and then, the subsequent oxidation of **56** to **10a** with O_2_/copper acetate occurred (Scheme 13) [108].

**Scheme 14:**
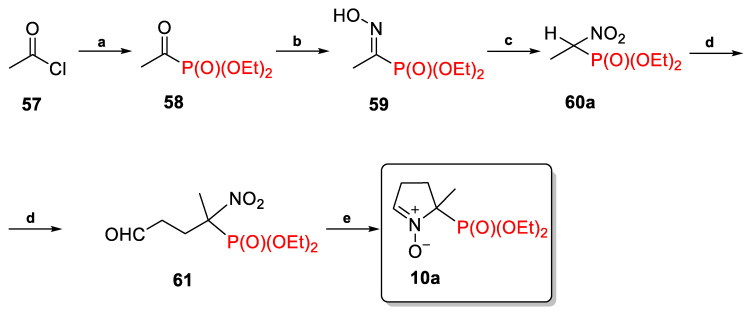
Synthesis of nitrone **10a**. Reagents and conditions: (a) P(O)(OEt)_3_, r.t., 24 h, 99%; (b) NH_2_OHxHCl, pyridine, EtOH, r.t., 12 h, 78%; (c) *m*-CPBA, CH_2_Cl_2_, r.t., 5 days, 76%; (d) acrolein, Et_3_N, MeCN, r.t., 2 h, 100%; (e) Zn/CH_3_COOH, ethanol/water (18:1), 10 °C, 1.5 h, then r.t., 1.5 h, 65%.

Another synthetic pathway to nitrone **10a** was based on the reductive cyclization of nitroaldehyde **61**. The synthesis of the respective intermediate **61** started with acetyl chloride (**57**) which was treated with triethyl phosphite to form α-ketophosphonate **58**. The subsequent reaction of **58** with hydroxylamine, followed by an oxidation reaction with acrolein, and a final reductive cyclization yielded the final product **10a** (Scheme 14) [94].

**Scheme 15:**
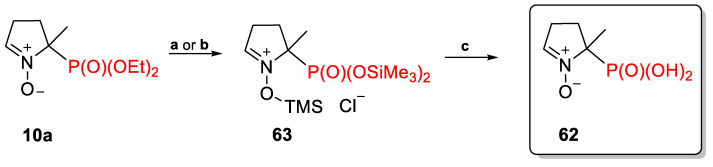
Synthesis of nitrone **62**. Reagents and conditions: (a) iodotrimethylsilane (3 equiv), CH_2_Cl_2_, r.t., 4.5 h; (b) bromotrimethylsilane (3 equiv), CH_2_Cl_2_, 30 °C, 20 h; (c) acetone, H_2_O, 76%.

Nitrone DEPMPO (**10a**), with a diethoxyphosphoryl function, was transformed into a phosphonic acid derivative **62** via a reaction with iodo- or bromotrimethylsilane followed by the hydrolysis of bistrimethylsilyl ester **63** (Scheme 15). The reaction was completed within 4.5 h at room temperature (r.t.) with iodotrimethylsilane, whereas the application of bromotrimethylsilane required increasing the temperature to 30 °C and extending the reaction time to 20 h [109].

**Scheme 16:**
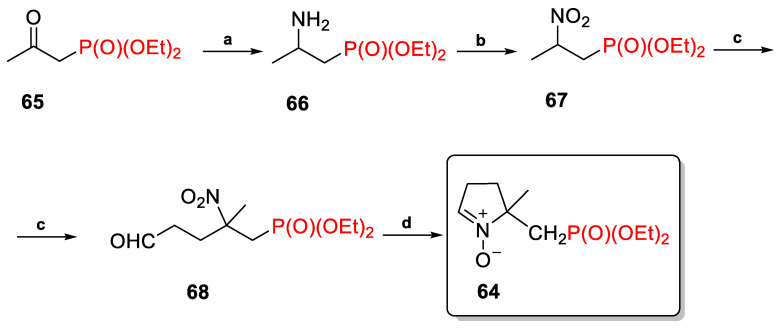
Synthesis of nitrone **64**. Reagents and conditions: (a) AcONH_4_, NaBH_3_CN, MeOH, r.t., 72 h, 86%; (b) *m*-CPBA, 1,2-dichloroethane, reflux, 16 h, 69%; (c) acrolein, MeCN, Triton B, 0 °C, 2 h, then 10 °C, 20 h, 64%; (d) Zn/AcOH, EtOH, 2 °C, 15 h, 30%.

For the synthesis of nitrone **64** functionalized with a diethoxyphosphorylmethyl substituent at C5, a methodology analogous to the synthesis of DEPMPO **10a** was applied, which relies on the reductive cyclization of nitroaldehyde **68** as a key step (Scheme 16) [110].

#### 2.3.2. Deuterium-Labeled Analogs of DEPMPO

**Scheme 17:**
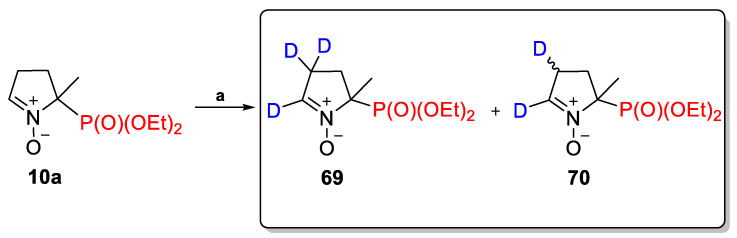
Synthesis of nitrones **69–70**. Reagents and conditions: (a) NaOD, D_2_O, 24 h, 16% (**69**/**70**: 80/20).

Deuterium-labeled phosphorylated cyclic nitrones **69**, **70**, **71**, **73,** and **79** [61,111] were designed with the intention of improving their properties as the spin traps for the detection of free radicals since the presence of deuterium should simplify the EPR spectra of the spin adducts [111]. The first attempt to synthesize deuterium-labeled analogs of DEPMPOs **69** and **70** involved the reaction of DEPMO (**10a**) with NaOD in D_2_O, leading to the formation of an inseparable mixture of 2,3,3-trideuterio nitrone **69** and 2,3-dideuterio nitrone **70** (Scheme 17) [61].

**Scheme 18:**
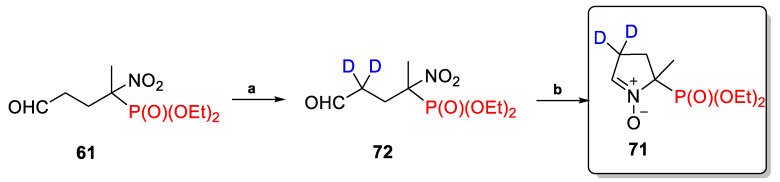
Synthesis of **71**. Reagents and conditions: (a) D_2_O, pyridine, r.t., 18 h, 84%; (b) Zn/CH_3_COOD in EtOD/D_2_O, 10 °C, 1.5 h, then r.t., 1.5 h, 79%.

For the synthesis of nitrone **71** with a deuterium located at C3, the reductive cyclization of the respective deuterated γ-nitroaldehyde **72** was used as a key step (Scheme 18). Compound **72** was prepared from the nitroaldehyde **61** [111] and obtained according to the procedure shown in Scheme 14 [94] using D_2_O for isotopic labeling (Scheme 18) [111].

**Scheme 19:**
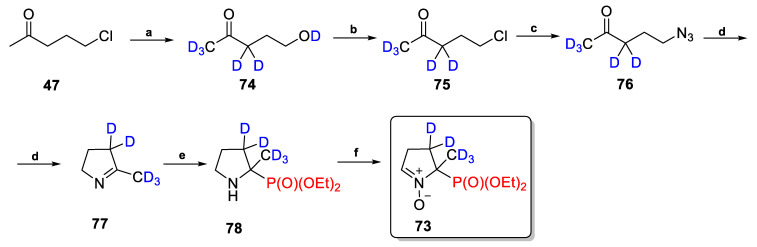
Synthesis of nitrone **73**. Reagents and conditions: (a) D_2_O, K_2_CO_3_, reflux, 15 h, 37%; (b) PPh_3_, CCl_4_, 80 °C, 30 min, 70%; (c) NaN_3_, *n*-Bu_4_NCl, DME, reflux, 2 h, 99%; (d) PPh_3_, Et_2_O, reflux, 4 h, 61%; (e) DP(O)(OEt)_2_, r.t., 7 days, 97%; (f) DMD, acetone, 0 °C, 2 h, 65%.

Pentadeuterated nitrone **73** was obtained from the 5-chloropentan-2-one (**47**). Isotopic labeling was accomplished via the treatment of **47** with D_2_O, and the resulting ketoalcohol **74** was then subjected to chlorination and azidation, followed by an aza-Wittig reaction to give 3,4-dihydro-2*H*-pyrrole derivative **77**. The reaction of **77** with deuterium-labeled diethyl phosphite followed by oxidation with DMD resulted in the formation of nitrone **73** (Scheme 19) [111].

**Scheme 20:**
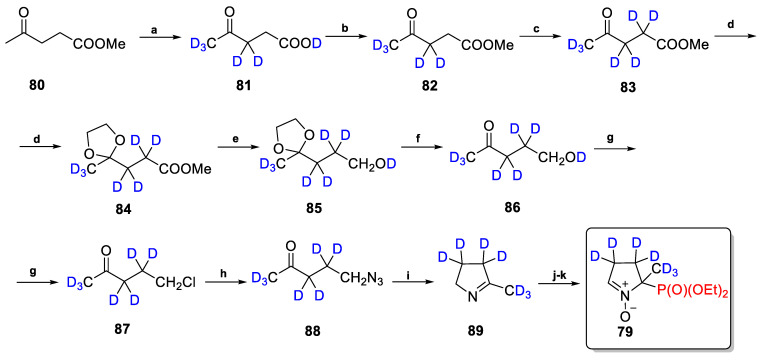
Synthesis of deuterated nitrone **79**. Reagents and conditions: (a) NaOD/D_2_O, 60 °C, 12 h, 92%; (b) CH_3_OD, D_2_SO_4_, reflux, 24 h, 75%; (c) Na/CH_3_OD, reflux, 1 h, 61%; (d) DOCH_2_-CH_2_OD, *p*-TsOH, benzene, reflux, 12 h, 69%; (e) LiAlH_4_, THF, reflux, 3 h, 97%; (f) DCl, D_2_O, THF, r.t., 12 h, 95%; (g) PPh_3_, CCl_4_, 80 °C, 30 min, 68%; (h) NaN_3_, *n*-Bu_4_NCl, DME, reflux, 2 h, 98%; (i) PPh_3_, Et_2_O, reflux, 4 h, 65%; (j) DP(O)(OEt)_2_, r.t., 7 days, 96%; (k) DMD, acetone, 0 °C, 2 h, 63%.

The synthesis of the perdeuterated analog of DEPMPO **79** was carried out starting from methyl 4-oxopentanoate **80**, which was then converted to the respective deuterated 3,4-dihydro-2*H*-pyrrole intermediate **89** via the reaction sequence shown in Scheme 20 [111].

#### 2.3.3. C3-Substituted Derivatives of DEPMPO

**Scheme 21:**
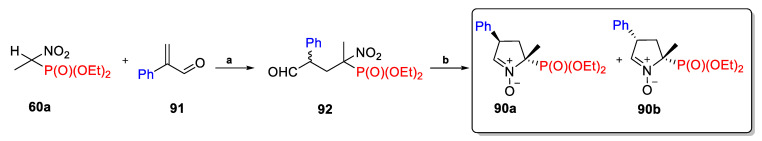
Synthesis of nitrones **90a**,**b**. Reagents and conditions: (a) Et_3_N, MeCN, r.t., 2 h, 99%; (b) Zn/NH_4_Cl, THF/H_2_O, 0 °C, 1 h, then r.t., 6 h, **90a**: 27%, **90b**: 25%.

**Scheme 22:**
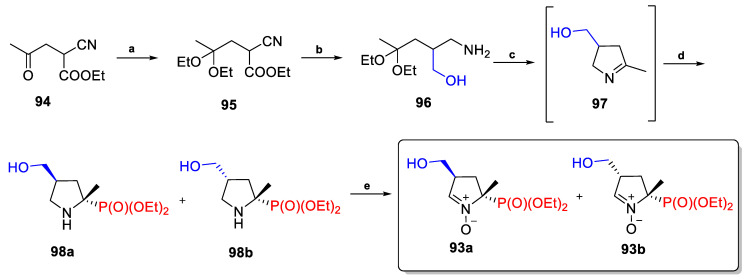
Synthesis of nitrones **93a**,**b**. Reagents and conditions: (a) Amberlyst 15, HC(OEt)_3_, CH_2_Cl_2_, 0–5 °C, 20 h, 99%; (b) LiAlH_4_, THF, 25 °C, 3 h, then 57 °C, 1 h, 36%; (c) 1. 5% HCl, THF/H_2_O (5/1), r.t., 3 h; 2. K_2_CO_3_, THF/H_2_O (5/1), r.t., 2 h; (d) HP(O)(OEt)_2_, r.t., 7 days, 40% (**98a**: 97%, **98b**: 3%); (e) H_2_O_2_/Na_2_WO_4_, MeOH/H_2_O (2.1), 4 °C, 48 h, 32% (**93a**: 97%, **93b**: 3%).

Two phosphorylated nitrones functionalized at the C3 position have been synthesized so far, namely **90** and **93** (Scheme 21 and Scheme 22, respectively) [103,104]. Two diastereoisomers of nitrone **90** with a phenyl group at C3 were prepared from nitrophosphonate **60a** [104] in a two-step reaction sequence involving the 1,4-addition of 2-phenylpropenal **91** with the formation of an inseparable mixture of aldehydes **92** followed by reductive cyclization to nitrones **90a** and **90b** (Scheme 21) [104].

Nitrones **93** functionalized with a hydroxymethyl group at C3 were efficiently synthesized via the oxidation of the respective diastereoisomeric diethyl (4-hydroxymethyl-2-methylpyrrolidin-2-yl)phosphonates **98** formed via the addition of diethyl phosphite to 4-hydroxymethyl-2-methyl-1-pyrroline (**97**). The synthesis of the key intermediate **97** was complex and started with the transacetalization of ethyl 2-cyano-4-oxopentanoate **94** with triethyl orthoformate, followed by the reduction of cyano and ester groups in **95** and the subsequent cyclization of aminoalcohole **96** to 2-methyl-1-pyrroline **97**. Imine **97** without purification was transformed into an inseparable diastereoisomeric mixture of pyrrolidines **98a** and **98b**, followed by oxidation with Na_2_WO_4_/H_2_O_2_ to form the final nitrones **93a** and **93b** [103].

#### 2.3.4. C4-Substituted Derivatives of DEPMPO

**Scheme 23:**
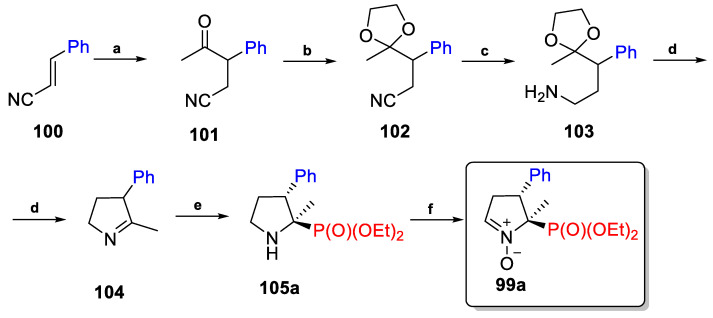
Synthesis of nitrone **99a**. Reagents and conditions: (a) Mg, Ac_2_O-TMSCl, DMF, 5–10 °C, 15 h, 76%; (b) camphorsulfonic acid, ethylene glycol, toluene, reflux, 8 h, 91%; (c) LiAlH_4_, THF, 0 °C, 2 h, then r.t., 7 h, 85%; (d) 3M HCl, THF/H_2_O, 4 h, then K_2_CO_3_, 1 h, 93%; (e) HP(O)(OEt)_2_ (**48a**), BF_3_•OEt_2_, r.t., 7 days, 54%; (f) Na_2_WO_4_/H_2_O_2_, EtOH/H_2_O, 0 °C, 48 h, 90%.

DEPMPO analogs functionalized at C4 have been synthesized to improve the properties of spin adducts with free radicals. Spectra of the spin adduct of standard cyclic phosphorylated nitrones such as DEPMPO (**10a**) and its analog with a diisopropoxyphosphoryl group with free radicals are complex due to the formation of two isomers of the spin adduct with free radicals [106]. The introduction of a substituent at C3, for example, a phenyl group, limited ring pseudorotation, but the half-life time of the spin adduct was worse than that of the spin adduct of DEPMPO (**10a**) [104]. Based on previous works, a new derivative of DEPMPO with a phenyl substituent at C4 was designed to improve the properties of the spin adduct [106]. Hardy and co-workers developed a method for the synthesis of DEPMPO analog **99** with a phenyl group at C4 (Scheme 23). To regiospecifically obtain a single isomer with the two largest *trans*-oriented substituents, i.e., diethoxyphosphoryl and phenyl groups, the synthetic pathway involving the application of pyrrolidine as an intermediate was developed [106]. Thus, cinnamonitrile (**100**) was subjected to a four-step reaction sequence including the *C*-acylation of α,β-unsaturated nitrile **100** promoted by Mg [106,112], the protection of the ketone group in **101**, the reduction of the CN group by LiAlH_4_, and the deprotection of the ketone group followed by the intramolecular cyclization to pyrroline **104**. Pyrroline **104** was then reacted with diethyl phosphite (**48a**) to afford pyrrolidine derivative **105a** and finally oxidized to nitrone **99a** (Scheme 23) [106].

**Scheme 24:**
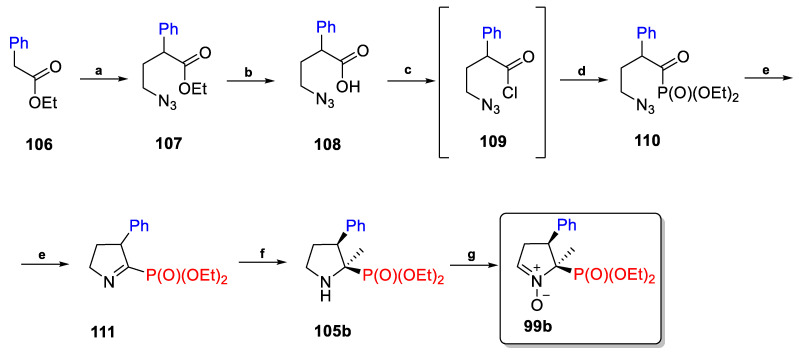
Synthesis of nitrone **99b**. Reagents and conditions: (a) LDA, THF, −78 °C, 1h, then 1-azido-2-iodoethane, r.t., 12 h, 59%; (b) 1M NaOH, EtOH, r.t., 3 h, 100%; (c) (COCl)_2_, CH_2_Cl_2_, r.t., 1 h, then reflux, 2 h; (d) P(OEt)_3_, CH_2_Cl_2_, r.t., 15 h, 100% (for steps c-d); (e) PPh_3_, Et_2_O, r.t., 16 h, 95%; (f) MeMgBr, BF_3_•OEt_2_, −78 °C, 4 h, 76%; (g) Na_2_WO_4_/H_2_O_2_, EtOH/H_2_O, 0 °C, 50 h, 64%.

The synthetic strategy for the preparation of isomeric nitrone **99b** with a *cis* configuration between diethoxyphosphoryl and phenyl groups began with the alkylation of ethyl phenylacetate **106** with 1-azido-2-iodoethane to give azidoester **107**, which was transformed into the acid chloride **109** via alkaline hydrolysis and a reaction with oxalyl chloride. The Arbuzov reaction of the crude chloride **109** led to the formation of (azidoacyl)phosphonate **110**, which subsequently reacted with PPh_3_ to give the corresponding 5-(diethoxyphosphoryl)pyrroline intermediate **111**. Finally, the designed nitrone **99b** was obtained from **111** via the addition of a Grignard reagent, followed by the oxidation of the resulting pyrrolidine derivative **105b** (Scheme 24) [106].

**Scheme 25:**
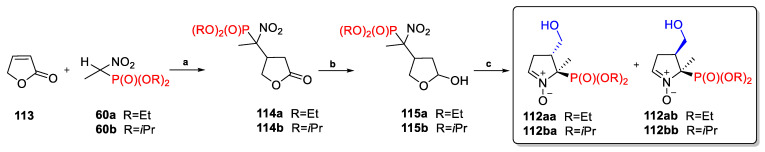
Synthesis of nitrones **112aa-bb**. Reagents and conditions: (a) PBu_3_, cyclohexane: CH_2_Cl_2_ (10:1), r.t., 66 h, 74–80%; (b) 1M DIBAL-H, hexane/CH_2_Cl_2_, −78 °C, 3 h, 50–75%; (c) Zn/NH_4_Cl, THF:H_2_O (10:1), r.t., 6 h, 60–64% as two diastereoisomers.

2(5*H*)-Furanone **113** was applied as the starting material for the synthesis of analogous nitrones **112** having a hydroxymethyl group at C4 in the pyrrolidine scaffold. Treatment of **113** with the respective carbanion obtained from nitrophosphonates **60a-b** gave derivatives **114a-b**, which were subsequently subjected to the reduction of carbonyl functions to form the final nitrones **112a**-**b** as the respective mixtures of diastereoisomers, which were successfully separated by column chromatography (Scheme 25) [105,107,113].

**Scheme 26:**
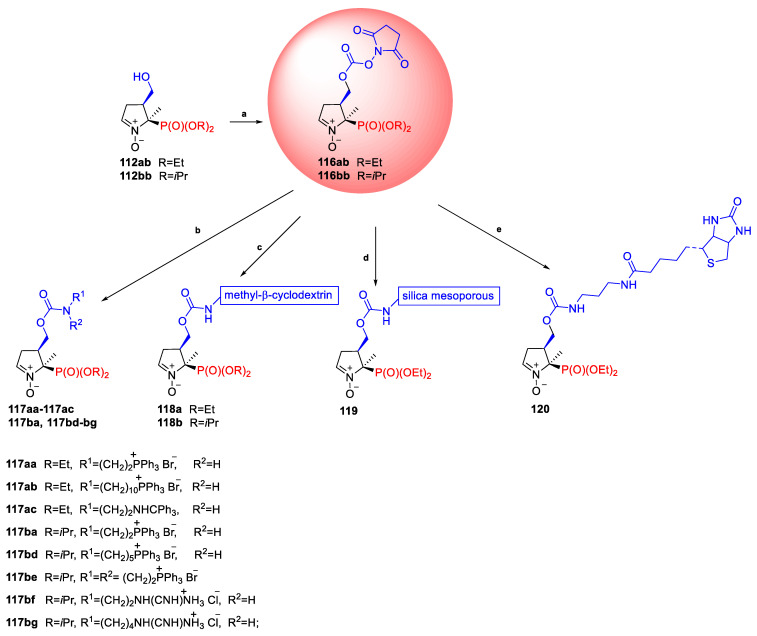
Synthesis of nitrones **116**, **117**, **118**, **119,** and **120**. Reagents and conditions: (a) disuccinimide carbonate, Et_3_N, MeCN, r.t., 36 h, 95%; (b) R^ʹ^R^ʺ^NH, Et_3_N, CH_2_Cl_2_, 2–18 h, 50–91%; (c) TRIMEB-NH_2_×HCl, Et_3_N, CH_2_Cl_2_, r.t., 80%; (d) SBA_15_-NH_2_, MeCN, Et_3_N, r.t., 12 h; (e) biotinylamidopropylammonium trifluoroacetate, Et_3_N, DMSO, r.t., 24 h, 39%.

While nitrones **112ab** and **112bb** with *cis* configurations between the dialkoxyphosphonyl and hydroxymethyl functions were efficiently transformed into respective succinimide carbonates **116ab** and **116bb** (Scheme 26) [105,107,113], the corresponding *trans*-isomer **112aa** proved less reactive, yielding small amounts of respective carbamates **116aa** (less than 20%) [105]. Cyclic nitrone **116ab** and **116bb** were applied as important precursors in further transformations, since the presence of the oxycarbonyloxyl group allowed for various functionalizations of a pyrrolidine scaffold at C4 via a substitution reaction [105,113], namely, the incorporation of triphenylphosphonium (**117aa-ac**, **117ba**, **117bd**, **117be**) [107,113,114,115,116] and guanidium groups (**117bf-bg**) [107], as well as β-cyclodextrin (**118a-b**) [114,117], mesoporous silica (**119**) [118], and biotin (**120**) [105,113] moieties (Scheme 26).

#### 2.3.5. C5-Modified Derivatives of DEPMPO

**Scheme 27:**
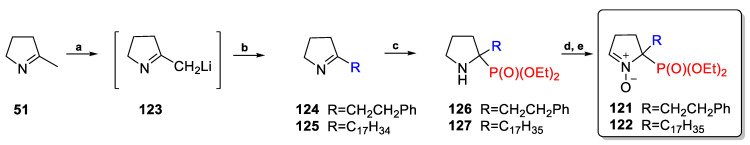
Synthesis of nitrones **121** and **122**. Reagents and conditions: (a) *n*-BuLi, THF, −78 °C; (b) 1-iodohexadecane, THF, −78 °C, 3 h, 60% for **125** or benzylbromide for **124**, −78 °C, 1 h, 68%; (c) HP(O)(OEt)_2_ (**48a**), r.t., 7 days, 90% for **126** or HP(O)(OEt)_2_, THF, reflux, 12 h, 80% for **127**; (d,e) *m*-CPBA, CHCl_3_, −10 °C, 1 h 35% for **121** or 3 h 15% for **122**.

DEPMPO derivatives **121** and **122**, with a phenylethyl and heptadecanyl group at C5, respectively, were synthesized from 2-methyl-1-pyrroline (**51**), which was transformed into corresponding functionalized 3,4-dihydro-2*H*-pyrrole derivatives **124** and **125**, followed by a reaction with diethyl phosphite (**48a**) and oxidation (Scheme 27) [100,102].

**Scheme 28:**
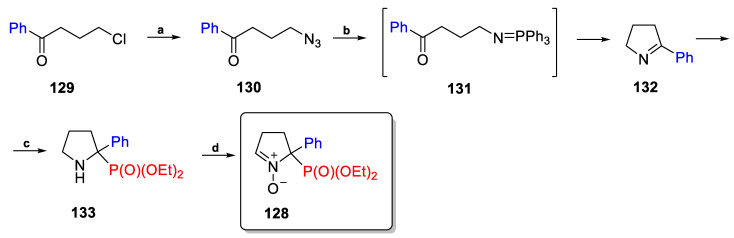
Synthesis of nitrone **128**. Reagents and conditions: (a) NaN_3_, tetrabutylammonium chloride, 1,2-dimethoxyethane, 75 °C, 16 h, (crude product); (b) PPh_3_, Et_2_O, pentane, r.t., 12 h, 68%; (c) HP(O)(OEt)_2_ (**48a**), BF_3_•Et_2_O, r.t., 24 h, 60%; (d) *m*-CPBA, CHCl_3_, −5 °C, 1 h, 25%.

The synthesis of DEPMPO derivative **128** required analogous 3,4-dihydro-2*H*-pyrrole **132** functionalized with phenyl at C5 prepared from 4-chloro-1-phenylbutan-1-one **129** via the reaction sequence depicted in Scheme 28 [101].

**Scheme 29:**
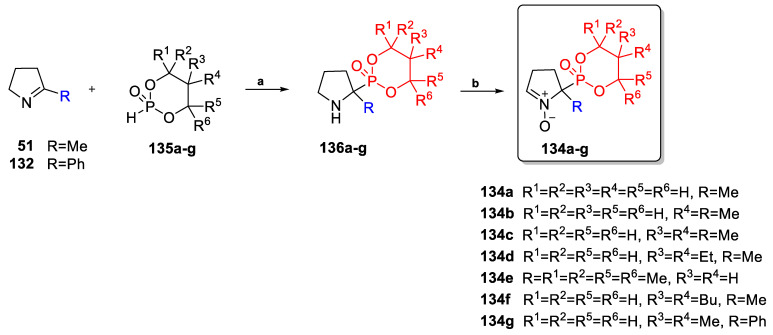
Synthesis of nitrones **134a-g**. Reagents and conditions: (a) toluene, r.t., 4–6 h or CH_2_Cl_2_, r.t.; (b) Na_2_WO_4_/H_2_O_2_, H_2_O_2_, 0 °C, 48 h or *m*-CPBA, CH_2_Cl_2_ or PSPO, 55–81%.

A series of nitrones **134a-g** containing the 1,3,2-dioxaphosphinane 2-oxide moiety, respectively, modified at C5 was prepared by a reaction of 2-methyl-1-pyrroline (**51**) or 2-phenyl-1-pyrroline (**132**) with cyclic phosphites **135a-g** followed by the oxidation of the obtained pyrrolidines **136a-g** (Scheme 29) [119,120,121].

**Scheme 30:**
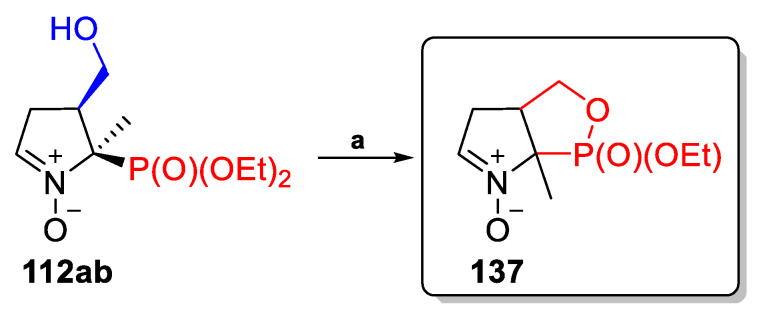
Synthesis of nitrone **137**. Reagents and conditions: (a) NaH, dimethoxyethane, r.t., 16 h, 45%.

Bicyclic nitrone **137** was prepared via the treatment of isomeric nitrone **112ab** with sodium hydride in the presence of dimethoxyethane (Scheme 30) [103].

#### 2.3.6. Analog of DEPMPO with Phosphoryl Group at C2

**Scheme 31:**
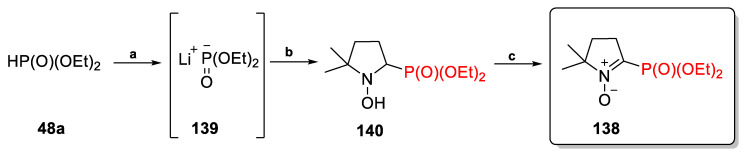
Synthesis of nitrone **138**. Reagents and conditions: (a) LDA, CH_2_Cl_2_, −10 °C, 15 min; (b) DMPO (**9**), CH_2_Cl_2_, −60 to −20 °C; 3.5 h, then H_2_O; (c) Cu(OAc)_2_×H_2_O, 29% NH_4_OH, EtOH, 10 min., 29%.

The addition of lithium diethyl phosphonate **139** to DMPO (**9**) and the subsequent oxidation of the resulting (1-hydroxypyrrolidin-2-yl)phosphonate **140** gave nitrone **138** decorated with diethoxyphosphoryl functions at C2 in the pyrrolidine ring (Scheme 31) [122].

#### 2.3.7. Diphosphorylated Derivative of DEPMPO

**Scheme 32:**
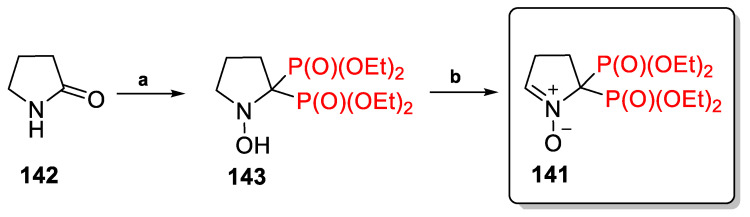
Synthesis of nitrone **141**. Reagents and conditions: (a) P(O)(OEt)_3_, POCl_3_, r.t., 5 h, then 32% NH_4_OH, 47%; (b) Oxone, tetrabutylammonium hydrogenosulfate, 0.1 M Na_2_HPO_4_ buffer, NaOH, acetone, CH_2_Cl_2_, 0 °C, 1 h, 11%.

The synthesis of bisphosphorylated analog **141** was achieved via the reaction of pyrrolidin-2-one (**142**) with triethylphosphite promoted by phosphoryl chloride, followed by the oxidation of the resulting (1-hydroxypyrrolidine-2,2-diyl)bis(phosphonate) (**143**) (Scheme 32) [123].

## 3. Detection of Free Radicals

**Figure 7:**
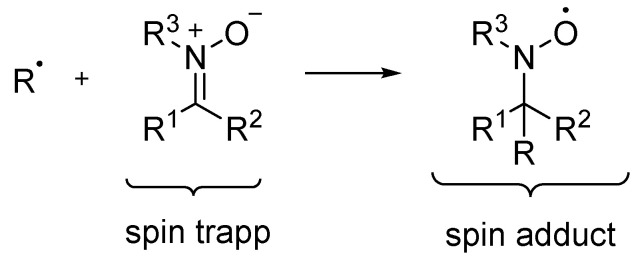
The reaction of a spin trap with radicals leading to a spin adduct.

Free radicals are chemical species with an unpaired electron and are therefore highly reactive [43,44]. They are ubiquitous in the living organism and have numerous functions to ensure their proper functioning [46,47,48,49,50,124]. Unfortunately, the overproduction of free radicals is harmful and causes the occurrence of many diseases [46,51,52,53,54,55,56,57,58,59]. The presence of ROS and RNS in both physiological processes makes it extremely important to find a suitable tool to detect these chemical species. One of the most useful methods is electron paramagnetic resonance (EPR). The main problem in detecting ROS and RNS is the short free-radical lifetimes (10^-3^ to 10^-9^ s) [125], which makes the direct detection of free radicals difficult. Admittedly, methods have been developed to detect free radicals alone, but they require the presence of TiO_2_ [126] or metal ions such as Ti^3+^ [127]. These methods cannot be used in biological systems because the presence of ion metals can interfere with the natural process of the cell. Currently, the best solution is to convert free radicals into more stable paramagnetic compounds to be detected by EPR techniques. The method involves the reaction of unstable and short-lived free radicals with diamagnetic (nitrones or hydroxylamines) or paramagnetic probes (nitroxides or trityl radicals), leading to the formation of further stable and long-living radicals. The nitrones most commonly used in EPR are called spin traps, and the more stable product of their reactions with free radicals are called spin adducts (Figure 7). For this reason, the technique is called EPR spin trapping and allows the concentration and identification of radicals formed in a biological system. EPR spin trapping has an advantage over other techniques because by analyzing spectral profiles of spin adducts with various radicals, they can be easily distinguished [9,128]. The main parameters of spectra are g factors and hyperfine splittings [129]. The spin trap should react rapidly with free radicals, the spin adduct formed should have a high stability, and its EPR spectra should be easy to interpret [93].

Spin traps can be divided into non-cyclic nitrones and cyclic nitrones [10,128]. PBN (**7**) and DMPO (**9**) (Figure 3) are the best studied, but they have some limitations that disqualify them. The spin adduct of PBN (**7**), like DMPO (**9**), has a very short half-life compared to its phosphorylated analogs [10,60,130]. The half-life of the hydroxyl radical spin adduct of PBN (**7**) in an aqueous solution was shown to be 90 s at pH 6 and even < 1 s for a pH above 9 [131]. Furthermore, the spin adduct of DMPO (**9**) with superoxide decomposed to a paramagnetic product that interfered with EPR spectra [130,132]. It is also worth mentioning that PBN (**7**) with ^•^OH and H_2_O formed indistinguishable EPR spectra [60]. DMPO (**9**) is also highly hydrophilic, suggesting that it cannot be used in every environment [61]. PBN (**7**) and DMPO (**9**) have many advantages; however, due to their limitations, they are not suitable for biological studies as spin traps. Therefore, it seemed necessary to modify their structure in such a way as to improve the properties of nitrones as spin traps. One of the best modifications was the introduction of a diethoxyphosphoryl group into the structure of PBN (**7**) and DMPO (**9**). The first “non-cyclic” phosphorylated nitrone was PPN (**8a**) (Figure 3) [60], while the first cyclic phosphorylated nitrone was DEPMPO (**10a**) (Figure 3) [61]. PPN (**8a**) was tested as a spin trap of the hydroxyl radical and hydroperoxyl radical. EPR spectra for hydroxyl spin adduct and hydroperoxyl were easy to distinguish, which was impossible when the spin trap was PBN (**7**) due to the presence of additional hyperfine splitting constants with phosphorus. Moreover, the presence of the diethoxyphosphoryl group improved the stability of the spin adducts of PPN (**8a**). Although the hydroxyl adduct partially decomposed, the presence of paramagnetic compounds did not interfere with the analysis of the EPR spectra [60]. The spin adducts of DEPMPO (**10a**) were persistent; for example, the superoxide spin adduct of DEPMPO (**10a**) was up to 15 times more persistent than DMPO (**9**) [61]. In addition, the reactions of DEPMPO (**10a**) with ^•^OH and O_2_^•^¯ are faster than with DMPO (**9**) (for DEPMPO-OH, an adduct was formed 2 times faster than the DMPO-OH adduct, and for DEPMPO-O_2_¯, an adduct was formed 1.5 times faster). Moreover, the superoxide spin adduct did not decompose like DMPO (**9**), and as in the case of PPN (**8a**), the presence of the phosphorus atom facilitates the identification of the EPR spectra of various radical spin adducts (presence of hyperfine splitting constants with phosphorus) [93]. PPN (**8a**) and DEPMPO (**10a**) were the first members of the new class of nitrones to show promising properties, but due to their high hydrophilicity, they could not be used in all environments [60,61]. Therefore, several phosphorylated analogs were synthesized and successfully used in both in vitro and in vivo studies.

### 3.1. “Non-Cyclic” Phosphorylated Nitrones

The largest group of “non-cyclic” phosphorylated nitrones are the PPN-type nitrones **8**. They are analogs of PPN (**8a**) (Figure 3) and differ in the type and position of the substituent on the aromatic ring. The introduced substituents influenced lipophilicity and enabled their application in various environments. This is a very important feature as it allows PPN-type nitrones **8** to be used in in vivo studies. Nitrones with Cl (**8c**), COOEt (**8w**), or Me (**8i**) groups (Figure 4) have been shown to have higher lipophilicity than PPN (**8a**), but the hydroxyl group increases the hydrophilicity of the respective nitrones **8j-o** [60,73,80,81,82,86,87,88]. Another way of altering lipophilicity was to functionalize PPN-type nitrones **8** with other moieties, such as glutathione [90] or a carbohydrate [91], to give the corresponding nitrones **38** (Scheme 10) and **37** (Scheme 9), respectively. A further modification of PPN (**8a**) was achieved by introducing additional nitrone functions. Thus, all polyphosphorylated nitrones **24a-d** (Scheme 12) were more lipophilic than PPN (**8a**). It was observed that the more nitrone functions, the better lipophilicity, with **24a** even having more than 30 times the lipophilicity of PPN (**8a**) (one nitrone function) [85]. The high lipophilicity likely influences the crossing of biomembranes [85]. PPN-type nitrones **8** are an effective spin trap because they can form spin adducts with various radicals and, most importantly, their EPR spectra can be easily distinguished. The presence of a phosphorus atom causes the formation of additional hyperfine splitting constants, which facilitates the analysis of the EPR spectra of the spin adducts and makes it easier to distinguish between radicals [60,73,80,81,82,86,87,88]. Even nitrones with a diphenylphosphinyl group could form stable spin adducts with radicals and, like PPN-type nitrone **8**, were easily distinguished [84].

As mentioned earlier, the detection of free radicals is extremely important to accurately study the effects of ROS and RNS during the physiological and pathological processes. However, the half-lives of free radicals are very short, and their concentrations are low [9]; thus, it is necessary to use spin traps targeting a specific location in the cells. Unfortunately, although PPN-type nitrones **8** and nitrones with diphenylphosphinyl groups are useful spin traps, they do not provide information about where the free radicals are produced in the cell. So far, only a few “non-cyclic” phosphorylated spin traps that target a specific side have been synthesized [90,91,133]. The incorporation of the glutathione molecule into the structure of the nitrone made GS-PPN **38** a dual-function free radical probe. Nitrone **38** is an efficient spin trap that captures free radicals and forms stable spin adducts. However, unlike previously presented nitrones, compound **38** was capable of scavenging O_2_^•^ ¯ generated in the PSII membranes of chloroplasts [90], while the glycosidic nitrone **37** was recognized by yeast lectins and had moderate scavenging properties [91,133].

### 3.2. Cyclic Phosphorylated Nitrones

Cyclic phosphorylated nitrones are also used as a spin trap. All nitrones, syntheses of which were presented in the previous chapter, were tested as potential spin traps. The first representative of this class of compounds was DEPMPO (**10a**), which has many advantages over its non-phosphorylated analogs; however, this nitrone shows better solubility in water than in a lipid medium [61]. Numerous modifications have been made to improve its solubility in lipid environments. Firstly, the replacement of the diethoxyphosphoryl group with another dialkoxyphosphoryl substituent has led to a huge number of analogs of DEPMPO (**10a**) [61,93,94,95,96,98,99]. All nitrones **10b-n** were capable of scavenging free radicals. However, various phosphoryl groups affected the lipophilicity of the spin trap, making nitrones **10b-n** more lipophilic than DEPMPO (**10a**) [93,94,96,98,99], which is a big advantage as the high lipophilicity improved cell membrane permeability [95]. In addition, nitrone **10b**, with a diphenylphosphinyl group, was also extremely stable (even after storing in a water solution at r.t. for several months, it did not decompose) [95]. On the other hand, the spin adduct of nitrone **62** (having a P(O)(OH)_2_ group) with superoxide was stable only in a pyridine solution, while its decomposition into another spin adduct in water was observed [109]. Replacing the hydrogen atoms with their isotope, i.e., deuterium in nitrones **71**, **73,** and **79**, simplified the analysis of the EPR spectra. In the case of nitrone **71** and **79**, the EPR spectra of the spin adduct with the *tert*-butylperoxyl radical were easy to interpret, as the two couplings disappeared when the α hydrogen was replaced by ^2^D. Moreover, the signal intensity of the EPR spectrum of the spin adduct of nitrone **79** with seven deuteriums was higher [111]. It also appeared that replacing the methyl group with another alkyl or phenyl group at C5 alters lipophilicity. The presence of a phenyl and phenylethyl substituent improved the solubility of nitrones **128** and **122** in a lipid environment [101,102], while nitrone **121** with a long alkyl chain was an amphiphilic spin trap. Due to its amphiphilic character, nitrone **121** is a site-specific spin trap and can detect free radicals on the PSII membrane [100]. Other site-specific spin traps have been synthesized, the most important being nitrone with biotin **120** [105,113] and a triphenylphosphonium cation **117aa-ab** as well as **117ba** and **117bd-be** [107,113,114,115,116]. Nitrone **120** is a specific spin trap due to the presence of biotin, which can form a complex with avidin [105,134]. Nitrones **117aa-ab**, as well as **117ba** and **117bd-be**, formed stable spin adducts with superoxide and, due to the presence of a triphenylphosphonium cation, targeted mitochondria [107,113,114,115,116]. Another important feature that spin traps have is their high resistance to the bioreduction process. Nitrones with β-cyclodextrin **118a-b** were less susceptible to degradation by heme proteins and biological reducing agents but could only be used to detect extracellular superoxide due to their high solubility [114,115]. Nitrone **119** with mesoporous silica exhibited promising properties. Above all, the detected superoxide anion in the biological system was highly stable, as nitrone **119** was resistant to GSH. It is worth mentioning that the stability of the spin adduct of **119** with a superoxide is higher compared to other spin traps [118]. The last class of nitrones that were used as spin traps are compounds **134a-g**, in which the phosphorous is a part of the six-membered ring. These were characterized by low cytotoxicity on the cell lines used and high lipophilicity. They also formed a stable spin adduct with superoxide. In addition, a mixture of cis/trans spin adduct diastereosomes was formed during the reaction of the chiral spin trap **134a-g** with the hydroxyl radical. Their EPR spectra provided information on the mechanism of ROS formation in the biological system [119,121].

## 4. Pharmacological Activity

**Figure 8:**
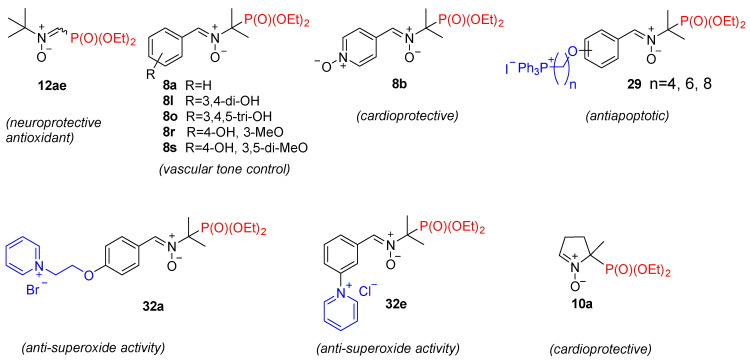
Examples of pharmacologically active phosphorylated nitrones.

Although many review papers on the pharmacological activity of nitrones have been published [75], these do not address the pharmacological use of phosphorylated nitrones. To date, phosphorylated nitrones have mainly been investigated for their antioxidant and neuroprotective properties. Thus, among all tested *N*-substituted *C*-phosphorylated nitrones **12aa-bb** (Figure 5, Scheme 1) screened for neuroprotective power, compounds **12ae** (Figure 8) were identified as the most potent and nontoxic agent with a high activity against neuronal necrotic cell death. Moreover, the obtained results correlate well with its great capacity for the inhibition of superoxide production (72%), the inhibition of lipid peroxidation (62%), and the 5-lipoxygenase (LOX) (45%) [74].

The effect of two PBN-derived phosphorylated nitrones **8a** and **8b** (Figure 8) on post-ischemic ventricular arrhythmias was investigated, demonstrating only limited antiarrhythmic cardioprotective effects during reperfusion after regional myocardial ischemia but also demonstrating minor antioxidant properties [135]. A series of PPN-type nitrones **8** (Scheme 6) with various substituted aromatic rings constructed on a natural phenolic antioxidant scaffold were also designed and tested for their antioxidant and NO-donating properties. Moreover, their cytotoxicity, vasorelaxant effect, and properties against ROS-induced vascular protein oxidation were also screened. Among them, nitrones **8l**, **8o**, **8r**, and **8s** (Figure 8), with the respective aromatic units structurally related to that of coffeic, gallic, ferulic, and sinapic acids, were found to be nontoxic, moderately lipophilic potential alternatives to PBN in pharmacological studies of vascular tone control [73]. Inspired by the antioxidant properties of nitrones **8**, a new series of functionalized triphenylphosphonium salt derivatives **29** (mito-PPNs) (Figure 8) were obtained by incorporating a lipophilic cation as a mitochondrial vector, assuming that the resulting compounds will be able to easily cross the mitochondrial inner membrane by diffusion. Indeed, nitrone **29** vectorized by a triphenylphosphonium cation appeared to be significantly more efficient against apoptosis in the selected cell type than non-cationic derivatives **30** with a diphenylphosphinyl group (Scheme 7) (75% of apoptosis inhibition for **29** vs. 55% in the case of compounds **30**) [83]. Further studies on mito-PPNs showed that a series of nitrones **31–33** vectorized by triethylammonium, pyridinium, and berberinium cations, respectively (Scheme 8), have the capacity to quench superoxides in vitro and increase the spin-trapping efficiency of biologically relevant free radicals, including superoxide and hydroxyl radicals. Their mitochondrial penetration was confirmed, and their antiapoptotic properties were assessed. Thus, two hydrophilic and nontoxic pyridinium-vectorized nitrones **32a** and **32e** (Figure 8) were selected as potentially better alternatives to triphenylphosphonium salt derivatives **29** for studying mitochondrial oxidative damage. As the authors stated, these nitrones showed good anti-superoxide activity that, combined with their fast intra-mitochondrial uptake and good EPR spin-trapping properties, could make them promising probes for further in vivo studies of mitochondrion-related pathological mechanisms involving ROS [89].

Cyclic phosphorylated nitrones are mainly used as spin traps [61,93,95,105,106,110,114]; however, the pharmacological properties of DEPMPO **10a** were also investigated [136,137,138,139,140]. Thus, the cardioprotective properties of DEPMPO **10a** were examined. It was found that DEPMPO pretreatment improves the recovery of cardiac function with the concomitant inhibition of post-ischemic superoxide (O_2_**^•−^**) production [139,140].

## 5. Conclusions

This paper provides a comprehensive review of the synthesis and biological activity of phosphorylated nitrones in the group of both non-cyclic and cyclic derivatives. While standard protocols are applied for the incorporation of nitrone function, major efforts were made to develop the strategies of introducing the phosphoryl function into the structure of target compounds and modification of their structures by installing various substituents within aromatic units leading to the preparation of PPN-type nitrones as well as within pyrroline scaffolds to obtain various cyclic nitrones as DEPMPO derivatives.

Both non-cyclic and cyclic phosphorylated nitrones are mainly used as spin traps, while their pharmacological properties are less recognized. Although the antioxidant and neuroprotective activities of selected PPN-type nitrones have been proven, including compounds of series **8**, **12**, and **29**, among the cyclic derivatives, nitrone DEPMPO (**10a**) has been mainly studied for its pharmacological properties, revealing its cardioprotective power.

## Data Availability

Data are contained within the article.
